# Executive Functioning in Schizophrenia

**DOI:** 10.3389/fpsyt.2013.00035

**Published:** 2013-06-24

**Authors:** Gricel Orellana, Andrea Slachevsky

**Affiliations:** ^1^Departamento de Psiquiatría Oriente, Facultad de Medicina, Universidad de Chile, Santiago, Chile; ^2^Servicio de Neurología, Hospital del Salvador, Santiago, Chile; ^3^Departamento de Neurología Oriente y Programa de Farmacología Molecular y Clínica, ICBM Facultad de Medicina, Universidad de Chile, Santiago, Chile; ^4^Centro de Investigación Avanzada en Educación, Universidad de Chile, Santiago, Chile; ^5^Departamento de Neurología, Clínica Alemana, Santiago, Chile

**Keywords:** control, schizophrenia, executive function, prefrontal cortex

## Abstract

The executive function (EF) is a set of abilities, which allows us to invoke voluntary control of our behavioral responses. These functions enable human beings to develop and carry out plans, make up analogies, obey social rules, solve problems, adapt to unexpected circumstances, do many tasks simultaneously, and locate episodes in time and place. EF includes divided attention and sustained attention, working memory (WM), set-shifting, flexibility, planning, and the regulation of goal directed behavior and can be defined as a brain function underlying the human faculty to act or think not only in reaction to external events but also in relation with internal goals and states. EF is mostly associated with dorsolateral prefrontal cortex (PFC). Besides EF, PFC is involved in self-regulation of behavior, i.e., the ability to regulate behavior according to internal goals and constraints, particularly in less structured situations. Self-regulation of behavior is subtended by ventral medial/orbital PFC. Impairment of EF is one of the most commonly observed deficits in schizophrenia through the various disease stages. Impairment in tasks measuring conceptualization, planning, cognitive flexibility, verbal fluency, ability to solve complex problems, and WM occur in schizophrenia. Disorders detected by executive tests are consistent with evidence from functional neuroimaging, which have shown PFC dysfunction in patients while performing these kinds of tasks. Schizophrenics also exhibit deficit in odor identifying, decision-making, and self-regulation of behavior suggesting dysfunction of the orbital PFC. However, impairment in executive tests is explained by dysfunction of prefronto-striato-thalamic, prefronto-parietal, and prefronto-temporal neural networks mainly. Disorders in EFs may be considered central facts with respect to schizophrenia and it has been suggested that negative symptoms may be explained by that executive dysfunction.

## Introduction

Schizophrenia affects approximately 1% of the world’s population. Generally, it begins in adolescence or the young adult stage, and lasts the patient’s lifetime. It is more frequent and severe in men. The disease is associated with significant psychosocial deterioration. Most who develop schizophrenia cannot return to work or finish their studies, and cannot establish normal social interactions. Additionally, 10% of patients with schizophrenia commit suicide. The treatments available in recent years reduce this suffering, but two thirds of patients require continued assistance from the public health system; thus, the monetary cost to society is enormous (Rossler et al., 2005).

This mental disease manifests with signs and symptoms that cover the entire range of human mental activity such as the ability to think creatively, to have close social relationships with other human beings, to use language and express ideas clearly, and to experience and express a variety of emotions. Schizophrenia has a multifactorial etiology, but these specific factors are currently unclear and the subject is a matter of intense research in various fields of neurobiology. Moreover, schizophrenia is considered as a neurodevelopmental illness (Schmitt et al., 2011). Efforts to identify the pathophysiology of schizophrenia currently focus on several lines of research: (i) neuroanatomical and neurofunctional abnormalities in the brains of patients, (ii) genes that confer susceptibility to schizophrenia and epigenomics studies, (iii) synaptic and immunological alterations, (iv) environmental risk factors, (v) neuropsychological disorders, and (vi) the mechanism of action of drugs that relieve symptoms.

Neuropsychological and neurocognitive paradigms are increasingly being used to identify dysfunctional structures and brain systems that underlie cognitive and behavioral disorders of schizophrenia (Heinrichs and Zakzanis, 1998). These paradigms implement experimental and clinical tests to better characterize neurocognitive abnormalities, and differ from previous psychological research in that they use functional neuroimaging with healthy controls or tests validated in populations with brain damage (Pantelis et al., 2002). Studying how patients with schizophrenia perform on neurocognitive tests has made it possible to identify central cognitive deficits that may explain a significant proportion of the social and vocational morbidity of this disease (Addington and Addington, 1999; Dickerson et al., 1999; Goldberg and Green, 2002).

This deficit in executive functions (EFs) may be central to schizophrenia and is present in adolescents at risk of developing the disease (ultra-high risk), in patients with a first outbreak of schizophrenia, and apparently in their first-degree relatives (Kuperberg and Heckers, 2000; Breton et al., 2011; Freedman and Brown, 2011). Mild to moderate impairments on EF tests have particularly been described in patients with a first-episode of schizophrenia (Flashman, 2002). In aged schizophrenic patients, a more severe cognitive impairment has been described that mainly involves EFs. Executive dysfunction has been significantly associated with psychosocial impairment in the disease. Despite its importance, there are still few studies that analyze the longitudinal course of executive functioning in schizophrenia (Flashman, 2002).

In this section, we will review the main changes in EF in schizophrenia. We will begin by defining EFs, their anatomical substrate, and the models of operation of the prefrontal cortex (PFC). Then, we will review the executive disorders described in schizophrenia. We will conclude by reviewing the neurocognitive models that attempt to explain those dysfunctions.

## Executive Functions and the Prefrontal Cortex

### Definition of executive functions and behavioral self-regulation

Prefrontal cortex lesions result in polymorphous symptomatology that can be grouped into four categories: cognitive, behavioral, emotional, and motivational impairments. Despite the polymorphism of the clinical manifestations, there are patterns of behavioral disorders and cognitive dysfunctions highly suggestive of frontal pathology such as the presence of overall hypoactivity associated with abulia, apathy, and lack of spontaneity, or conversely, global hyperactivity associated with distractibility, impulsivity, and disinhibition. Likewise, a syndrome characterized by excessive adherence to environmental stimuli can be observed: patients imitate the examiner’s gestures, although not instructed to do so (imitation behavior), and object presentation implies the order to grasp and use them (utilization behavior). Patients may also present perseverative and stereotyped behaviors. The presence of confabulation and reduplicative paramnesia, anosognosia, or anosodiaphoria, and disorders of emotion, social behavior, sexual behavior, micturition, and behavioral control also suggest frontal lobe dysfunction (Godefroy, 2003). The main cognitive manifestations that suggest prefrontal dysfunction are deficits in response initiation and suppression, focused attention, rule deduction, and problem solving as well as difficulties in planning, information generation, maintaining a response pattern, and changing a response pattern to another (Godefroy, 2003). Other related cognitive disorders are of sustained attention, task coordination, and divided attention, and related memory disorders are linked to a failure in the ability to encode and retrieve information as part of the strategic mnemonic process. Patients also exhibit disorders in planning for the future (Godefroy, 2003) and in social cognition expressed as difficulties in understanding the mental states of others and attributing intentions (i.e., theory of mind or mentalizing).

All these clinical manifestations correspond to either a “dysexecutive syndrome” reflecting disturbances of executive cognitive functions or a “self-regulatory disorder” (SRD) reflecting disturbances of behavioral/emotional regulatory functions (Stuss and Alexander, 2009). The concept of a cognitive executive system involves different processes that mainly function to allow an individual to adapt to new situations, especially when action routines (i.e., overlearned cognitive skills) become insufficient (Van der Linden et al., 2000). According to Lezak (1995), EFs correspond to the mental capabilities necessary for formulating an objective, planning, and implementing actions to achieve that objective, and then intervening in the performance of complex tasks (Dubois et al., 1994; Lezak, 1995). These functions act when behavior control processes are required and depend on three cognitive actions: shifting or changing among different tasks or mental sets, inhibiting irrelevant automatic responses, and updating mental representations held in working memory (WM) (Miyake et al., 2000; Van der Linden et al., 2000). The concept of self-regulation of behavior is the ability to regulate behavior according to internal goals and constraints (Goldberg and Podell, 2000). This arises from the ability to hold a mental representation of the self online and to use this information to inhibit inappropriate responses (Levine et al., 1998), implying the ability to modify behavior while taking the environment and the consequences of actions into account. SRD is most apparent in unstructured situations (e.g., childrearing, making a major purchase, or occupational decision-making) in which patients fail to inhibit inappropriate responses in favor of responses that might result in a preferential long-term outcome (Stuss and Levine, 2002). Albeit, others authors have proposed different nomenclature for the dysexecutive syndrome and the SRD. Chan et al. (2008) have proposed that both disorders are in a single executive disorder subdivided into both hot and cold component. Godefroy and Stuss (2007) proposed to denominate dysexecutive syndrome as a cognitive dysexecutive syndrome and the SRD as behavioral dysexecutive syndrome. In this review, we could refer these syndromes as cognitive dysexecutive syndrome and SRD.

### Prefrontal cortex neuroanatomy

**Figure 1:**
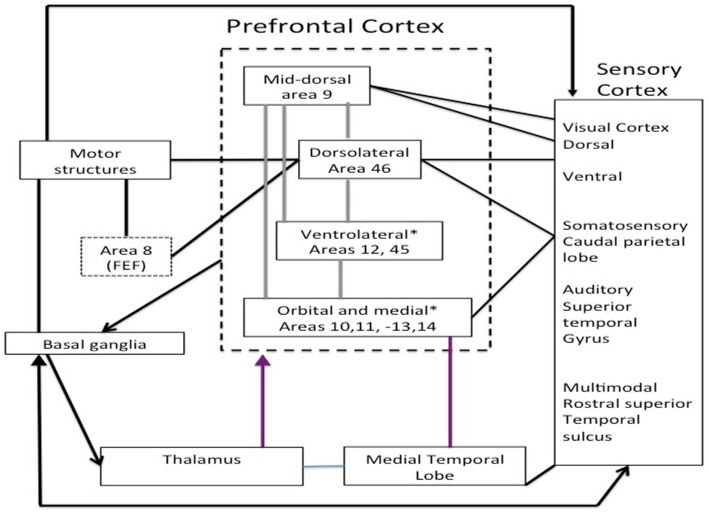
**Main regions of the prefrontal cortex adapted Miller and Cohen (2001)**.

Anatomy allows us to understand PFC intervention in EFs and the self-regulation of behavior. A proper understanding of PFC neuroanatomy is crucial to understanding models of its function. The frontal lobes can be divided into three functional sectors: (i) a motor and premotor sector, (ii) a paralimbic sector located in the ventral and medial sides of the frontal lobe, which consists of the anterior cingulate complex (areas 23 and 32), paraolfactory gyrus (area 25), and posterior orbitofrontal regions, and (iii) a heteromodal sector including areas 9, 10, 45, 46, and 47, and the anterior portion of areas 11 and 12. Of these three sectors, the paralimbic and heteromodal sectors constitute the PFC, which can be anatomically and functionally divided into the dorsolateral PFC (DLPFC) and orbitofrontal PFC, consisting of the frontal pole and the ventral PFC (VPFC) (Stuss and Levine, 2002; Slachevsky and Alegria, 2005; Slachevsky et al., 2005, 2009) (cf. Figure 1).

The main connections between these two functional regions allow us to understand their role in behavior. The VPFC receives input from the brainstem and the diencephalon that integrate information about the internal environment, awareness level, motivation, state, and neurocognitive manifestations of emotions. Within the VPFC, we can distinguish the ventromedial PFC, which receives inputs from the DLPFC and regions involved in emotional processing (amygdala), memory (hippocampus), and complex visual processing (temporal association cortex). The DLPFC maintains reciprocal connections with brain regions involved in motor control [basal ganglia, supplementary motor area (SMA), and premotor cortex], action monitoring (cingulum), and complex processing of sensory stimuli (temporal and parietal association cortices) (Barbas, 2000). An additional functional division is the superomedial prefrontal region, and lesions to this area result in an apathetic syndrome characterized by decreased initiative (Stuss and Levine, 2002). In summary, based on the connections of the PFC, four main regions can be identified: (i) the ventromedial PFC primarily involved in the integration of emotional information kept in memory and coming from the environment, (ii) the DLPFC involved in WM and the main EFs, (iii) the medial PFC, especially the superomedial area, involved in attentional control and planning, and (iv) the frontal pole involved in adaptive planning and self-awareness (Stuss and Levine, 2002) (cf. Figure 1; Table 1).

**Table 1:** **Functional and anatomical divisions of the prefrontal cortex adapted from Grafman and Litvan (1999)**.

Prefrontal cortical area	Cognitive domain	Neurobehavioral probe^1^
Dorsolateral	Working memory	Can the patient remember a telephone number after a very short pause?
	Reasoning	Can the patient explain how two objects are similar (e.g., a banana and an apple are both fruits), deduce an answer to a mystery, or adjust to an unforeseen demand or event?
	Thematic understanding	Can the patient read a short article or watch a brief television program and understand the point or theme of what they read or watched?
Ventromedial	Social skills	Does the patient make inappropriate sexual remarks, eat excessively, or disobey other typical social rules of behavior?
	Inhibition of prepotent responses	Does the patient exhibit stereotyped behaviors such as repeating the same phrase or activities over and over again?
	Motivation and reward	Does the patient still enjoy the same activities or items they used to?
Medial	Allocation of attention	Do irrelevant sounds or sights in the environment distract the patient?
	Predictive planning	Can the patient do routine activities, such as using an automated teller machine or using a kettle to make a cup of tea?
Frontopolar	Adaptive planning	Can the patient be interrupted in the middle of a conversation to answer the telephone and then, without cuing, resume the conversation appropriately after hanging up?

*^1^Compared with premorbid behavior*.

There are also reciprocal connections between the PFC and basal ganglia. These connections are organized in parallel and segregated prefrontosubcortical circuits. There are five circuits that originate from five different prefrontal regions: the DLPFC, orbitofrontal cortex (OFC), anterior cingulate cortex (ACC), frontal eye fields, and SMA.

### Models of prefrontal cortex function

Several models have conceptualized the role of the PFC. Luria (1980) systematized PFC participation in adaptive behaviors, breaking them down into four stages: initial data analysis, preparation of a program that organizes and directs the various activities necessary for task completion, program execution, and comparison of results with the initial data (Luria, 1980; Wood and Grafman, 2003). This model was carried out by Shallice (1982) who distinguished two main processes: (i) a repertoire of normal and relatively automatic motor and intellectual actions handling repetitive situations of everyday life, and (ii) a supervisory attentional system (SAS) that intervenes when a new or complex activity needs the development of strategies, planning of the different stages of an action, and inhibition of irrelevant responses (Shallice, 2002). The SAS may be located in the PFC (Shallice, 2002) and this function was clearly conceptualized by Mesulam (2002) (Koechlin et al., 2003). According to this researcher, the primitive brain may have had a default mode that causes a familiar environmental stimulus to trigger automatic and inflexible responses for immediate gratification. Therefore, the default mode would leave no room for prediction or modification of the stimulus-response association in relation to the external environment and individual experiences. The main physiological function of the PFC may thus be to remove and transcend this primitive mode of response, allowing for the generation of more flexible and contingent responses. In addition, this model postulates that the influence of the PFC is manifested through its core functions, namely: (i) WM, (ii) inhibition of distractibility, perseveration, and instant gratification, (iii) active search for novelty, (iv) emotional coloring of action, and (v) context coding, perspective taking, and understanding others (Mesulam, 2002). These processes allow for a switch from an inflexible to a flexible stimulus-response association that considers the context in which a stimulus is presented and individual experiences. Other theories have linked PFC function with WM processes that keep information online, perform cognitive operations on that information, and/or plan actions on that information (Goldman-Rakic, 1987). Along these lines, Fuster proposed that the PFC allows for temporal integration of behavior through the intervention of three cognitive processes: WM, inhibition, and preparation to achieve goals (Fuster, 2001).

While several different models have been proposed to explain the role of the PFC, Miyake et al. (2000) are of the opinion that none of these models specify the role of different cognitive functions or processes that enable cognitive control. Cognitive control refers to the ability to regulate thoughts and actions in accordance with internally represented behavioral goals (Braver, 2012). It allows information processing and behavior to vary adaptively from moment to moment depending on current goals rather than remain rigid and inflexible (Miyake et al., 2000). The functional organization of the PFC and modular architecture corresponding to cognitive control are still poorly understood (Slachevsky et al., 2009).

Three new models allow for a better understanding of the PFC’s role in cognitive control. Two of these models, those separately put forward by Badre and Koechlin, propose that the laterofrontal regions along the rostrocaudal or anterior-posterior axis interact with one another hierarchically (Koechlin et al., 2003; Badre, 2008; Badre et al., 2009). According to this model, there is a dominance relationship whereby higher, more anterior regions influence processing in lower, more posterior regions to a greater extent than vice versa. The third model, the dual mechanisms of control (DMC) framework, postulates that the intrinsic variability of cognitive control arises from qualitative distinctions in temporal dynamics between proactive and reactive modes of control instead of a hierarchical organization. Proactive control reflects the sustained and anticipatory maintenance of goal-relevant information within the lateral PFC (LPFC) to enable optimal cognitive performance, whereas reactive control reflects transient stimulus-driven goal reactivation that recruits the LPFC (plus a wider brain network) based on interference demands or episodic associations (Braver, 2012).

**Figure 2:**
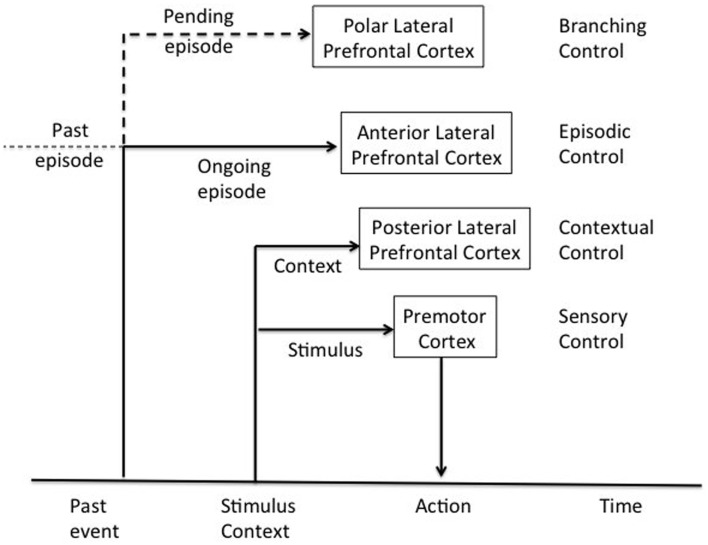
**Architecture of cognitive control of the human prefrontal cortex adapted from Koechlin et al. (2003)**.

Koechlin’s model postulates that the DLPFC is organized as a cascade of representations ranging from the premotor cortex, the more posterior, or caudal part of the DLPFC, to the more anterior or rostral regions of the DLPFC, the frontal pole (Koechlin and Summerfield, 2007). These various representations manage responses to the many different signals needed to control actions. In this cascade architecture, the recruitment of control processes from more posterior to more anterior zones depends on the temporal structure of the representations that relate actions to the signals that determine them. The model distinguishes four levels of action control (cf. Figure 2): (i) at the base of this cascade, the premotor cortex involved in *sensory control* and the selection of motor actions in response to stimuli; (ii) toward the ventral area, caudal regions of the DLPFC (Brodmann areas 9, 44, and 45) involved in *contextual control*-activation of premotor representations or stimulus-response associations depending on perceptual contextual cues that accompany stimulus appearance; (iii) rostral DLPFC regions involved in *episodic control*-activation of the aforementioned flow representations (tasks or a coherent set of stimulus-response associations evoked in a context) depending on the time course in which stimuli appear based on past events; and (iv) more anterior regions of the DLPFC, the frontal pole (Brodmann area 10) involved in *control of ramifications*-activation of rostral prefrontal representations (episodes of behavior or action plans) based on concomitant development of action plans. These different levels are given information about stimuli from posterior associative regions. Thus, the prefrontal regions receive information about stimuli and their external context, and the temporal events in which the stimuli occur. Given the anatomical connections of the PFC, the model postulates a cascade of control extending from anterior to posterior DLPFC regions, the latter controlled by the former. This cascade model has the great advantage of proposing a functional description of the PFC based on elementary cognitive processes, positing how these different processes are coordinated in the PFC. In other words, it explains the role of the LPFC in cognitive control and the anatomofunctional organization of the LPFC (Koechlin et al., 2003).

Badre and D’Esposito (2009) proposed that the rostrocaudal hierarchy in the DLPFC can be better understood in terms of differences in control demands, defined based on the form of the representations that compete during action selection. Action representations may be organized hierarchically such that more abstract action representations designate a set of more specific representations. For example, a task set can be labeled as abstract because it generalizes across a set of specific stimulus-response mappings. As representations at progressively more abstract levels compete, distinct control processors along the rostrocaudal axis of the PFC may resolve the competition (Badre and D’Esposito, 2009).

## Disorders of Executive Functions in Schizophrenia

Schizophrenia researchers observed cognitive abnormalities early on. Kraepelin commented in 1913 that “mental efficiency is always diminished to a considerable degree” and that “patients are distracted, inattentive, they cannot keep the thought in mind” (Kraepelin, 1913). Vigotsky (1934) used neuropsychological tests to argue that dysfunction in abstract or conceptual thinking was observed in schizophrenia. In the preneuroleptic decade, Rapaport reported that patients yielded results worse than controls on tests that assessed judgment, concentration, planning ability, and anticipation, and that there was deterioration in concept formation and memory. Hunt and Cofer (1944) then found that the intelligence quotient of schizophrenics was lower than that of controls. Neuropsychological studies have shown that the most prominent cognitive impairments exhibited by patients with schizophrenia include distractibility, loose associations, disorganized or socially inappropriate behavior, and disorders of EFs (Braver et al., 1999).

Recent studies have sought to characterize the prevalence, extent, and nature of cognitive disorders in schizophrenia. Impaired cognition across a wide range of cognitive domains is a pervasive feature of schizophrenia and is connected to poor functional outcome for patients (Blanchard et al., 2011; Fett et al., 2011). There is no general consensus as to whether the cognitive impairments seen in schizophrenia can be attributed to a single disrupted mechanism, to multiple disrupted systems, or to low-level perceptual deficits. Green argues that a generalized deficit, broadly defined, probably does not exist in schizophrenia (Green et al., 2013). Cognitive disorders in schizophrenia are heterogeneous, sometimes they are selective and specific, and manifested by different patterns of associated and dissociated performance on different cognitive tasks (Kuperberg and Heckers, 2000). Neuropsychological deficits are associated with psychosocial dysfunction, and are dissociated from psychiatric symptoms, global cognitive efficiency, and intelligence in schizophrenia (Addington and Addington, 1999; Dickerson et al., 1999; Goldberg and Green, 2002; Badcock et al., 2005; Kopald et al., 2012; Green et al., 2013).

Moreover, global measures of cognition capture a broad swath of abilities; they do not provide a depth of knowledge about any particular area, such as executive functioning (Freedman and Brown, 2011).

Disorders of EF are the most commonly observed cognitive deficits in schizophrenia. Negative schizophrenic symptoms are very similar to symptoms of patients with lesions of the dorsomedial PFC and related structures (Freedman and Brown, 2011). Schizophrenia patients show deficits in tasks related to the DLPFC. These disorders are manifested in motor programing due to difficulties in temporal and sensory information integration, planning and maintenance of goal-oriented behavior, and behavioral flexibility. These disorders can be objectified by neuropsychological tests that evaluate different skills: conceptualization, cognitive flexibility, ability to solve complex problems, and WM. Deficits in visuospatial and verbal WM are prominent in schizophrenia and fundamental to schizophrenia (Callicott et al., 2003).

Disturbances in cognitive flexibility have been measured with tasks such as the Wisconsin Card Sorting Test (WCST), the Trail Making Test (TMT) Part B, and verbal fluency tests (Ihara et al., 2000). The WCST measures conceptualization and cognitive flexibility. During the test, participants must shift attention among different dimensions of stimuli based on feedback. The WCST is particularly sensitive to lesions of the DLPFC and upper medial regions of the PFC (Pantelis et al., 2002). Importantly, reduced DLPFC gray matter volume is significantly more pronounced in schizophrenic patients with greater executive dysfunction as measured by the WCST (Eisenberg and Berma, 2010). However, the WCST should be used with caution as a frontal measure because retrorolandic cortex lesions, such as hippocampal lesions, have also been associated with decreased performance, specifically increased perseverative errors (Rossler et al., 2005). Chronic and first-episode schizophrenia (FES) patients present difficulties in inhibiting previously learned responses and fail to shift their attention to relevant stimuli, thus making perseverative errors. They perseverate on an answer previously mentioned as incorrect. It has been suggested that the diminished performance of schizophrenics reflects difficulty in inhibiting inappropriate responses.

Cognitive flexibility has also been assessed with the Cambridge Neuropsychological Test Automated Battery’s intradimensional (ID)/extradimensional (ED) task. The ID/ED task measures discrimination and reversal learning whereby the participant is required to shift responses based on rules that classify figures as “correct” or “incorrect.” In the first stages, the patient must learn to respond to rules that require shifting responses within one category of features in the display (the ID shift) from a previously reinforced lined figure to another lined figure. The last two stages (the ED shift) require shifting responses among categories (e.g., the patient must respond to colored shapes instead of lined figures). Unmedicated first*-*episode schizophrenic patients succeeded in shifting among categories (ED), whereas moderately severe schizophrenic patients failed in the ED category shift due to a tendency to persevere, similar to frontal patients. Patients with chronic schizophrenia failed in both ID and ED shifting. These studies suggest progressive deterioration in performance on this task, but further neuroimaging studies are required to confirm this finding (Pantelis et al., 2002).

Schizophrenia patients demonstrate poor performance on tasks that measure planning capacity as in the Tower of London. Recent research using dual-task paradigms have also provided evidence that patients are poor at performing two tasks simultaneously and alternating between two different tasks (Braver et al., 1999; Pantelis et al., 2002).

Koechlin’s model of cognitive control was recently evaluated in schizophrenics, and results showed that schizophrenic patients retained both sensory and episodic dimensions of cognitive control but presented great difficulty in contextual conditions as selecting the appropriate response required taking into account information related to the perceptual context. Contextual control can be considered a set of executive processes mediating the hierarchical organization of behavior. A deficit in cognitive control therefore reflects a specific problem in the hierarchical control of action, leading to selection of inappropriate behavioral representations for ongoing action plans. Impairment in contextual control was a good predictor of disorganization syndrome scores, suggesting that the impairment may result from a deficit in the combination or selection of hierarchically organized action representations (Chambon et al., 2008). Concerning the DMC, there is evidence of an association between impairments in the DLPFC and proactive control in schizophrenia for both medicated and unmedicated patients as well as those at risk of developing schizophrenia (Barch and Ceaser, 2012). To the best of our knowledge, Badre’s model of cognitive control has not been tested in schizophrenics.

In a recent meta-analysis about functional neuroanatomy of schizophrenia, Minzenberg et al., found that healthy adults and schizophrenic patients activate a qualitatively similar cortico-subcortical neural network during executive task performance, consistent with the engagement of a general-purpose cognitive control network, with critical nodes in the DLPFC and ACC. But, patients with schizophrenia show altered activity with deficits in the DLPFC, ACC, and mediodorsal nucleus of the thalamus. Increases in activity are evident in other PFC areas, which could be compensatory in nature (Minzenberg et al., 2009).

Though less studied in schizophrenia than the DLPFC, the VPFC may show less cellular abnormalities than the DLPFC (Eisenberg and Berma, 2010). Schizophrenic patients exhibited impairment on tests evaluating the VPFC: an emotional decision-making task, and tests assessing self-regulation. Furthermore, VPFC lesions lead to decreased ability to identify smells and a deficit in identifying smells has been demonstrated in schizophrenics (Pantelis et al., 2002). On the other hand, the study of decision-making has yielded contradictory results: one study with a low patient sample size showed that schizophrenics did not differ from controls, but a more recent study showed poorer performance in schizophrenics (Ritter et al., 2004).

Self-regulation of behavior is evaluated with the Six Elements Test, which requires participants to organize their activities in order to perform six tasks in a limited period of time while obeying a few rules. The participant carries out the task alone without feedback from the examiner (Evans et al., 1997). We reported that first-episode schizophrenics exhibited poorer performance than the control group in the Six Elements Test (Orellana et al., 2007; Orellana, 2009). Our results are consistent with other studies of chronic (Evans et al., 1997; Ihara et al., 2000; Jovanovski et al., 2007) and unmedicated first-episode schizophrenics, suggesting an “SRD” (Cheng and Chan, 2005; Chan et al., 2006; Liu et al., 2010).

One study suggested that executive dysfunction in first-episodic schizophrenics may be identified with rapid assessment tools that measure different PFC functions such as the Frontal Assessment Battery at bedside (FAB) (Dubois et al., 2000). Scores on the six subtests of the FAB were significantly correlated with frontal metabolism of patients with frontal damage in positron emission tomography (PET) studies (Dubois et al., 2000). Additionally, we reported that in comparison with controls, first-episode schizophrenics showed decreased performance on the conceptualization, verbal fluency, motor programing, sensitivity to interference, and inhibitory control subtests of the FAB (Orellana et al., 2007; Orellana, 2009).

Finally, it is important to note that normal performance on standard frontal neuropsychological tests, such as the WCST and TMT, does not negate executive dysfunction. Shallice and Burgess emphasize that in most neuropsychological tests, patients undergo short trials in which another person encourages task initiation and successful trial completion typically involves engaging in a single explicit problem at a time. This significantly differs from the demands of everyday life, which involve organizing or planning behavior over long periods of time, or setting priorities when faced with competing tasks (Shallice and Burgess, 1991).

Questionnaires have been designed to measure the impact of a dysexecutive syndrome in daily life and overcome the low sensitivity of standard neuropsychological tests. The Dysexecutive Questionnaire (DEX) of the Behavioral Assessment of the Dysexecutive Syndrome (Wilson et al., 1996; Burgess et al., 1998) and the Inventory of the Behavioral Dysexecutive Syndrome (IBDS) of the Groupe de Réflexion sur l’Évaluation des Fonctions Exécutives (Godefroy et al., 2010) evaluate the presence of behavioral disorders that reflect a dysexecutive syndrome in everyday life. As previously mentioned, this syndrome is characterized by deficits in frontal lobe control over behavior. These disorders are most obvious when patients try to handle daily life situations that are complex, open, and socially ambiguous. The DEX and IBDS have allowed for identification of behavioral disturbances associated with executive dysfunction in FES (Orellana et al., 2007; Orellana, 2009). Studies in chronic patients using the DEX have also showed statistical differences with controls (Evans et al., 1997).

## Executive Attention and Schizophrenia

Executive functions have been related to executive attention. Attention is not a unitary system but a set of integrated processes involved in all levels of cognitive processing from sensory input to motor output (Colmenero et al., 2001; Gitelman, 2003). These different processes depend on three different yet closely related neural networks: (i) alert, which underlies the ability to achieve and maintain a vigil and aware state, (ii) orientation to sensory events, which underlies the ability to select information from sensory inputs (i.e., selective attention), and (iii) executive control of thoughts and feelings (i.e., so-called executive attention) (Posner and Fan, 2009). Executive attention differs from EFs in that the latter encompasses different processes, whereas attention stresses the role of monitoring and resolving conflict among computations occurring in different brain areas, and can be measured with simple tests (Posner and Fan, 2009).

The Attention Network Test (ANT), a mental chronometry test, can evaluate the three attentional networks, providing a measure of the effectiveness of each (Fan et al., 2002). A study using the ANT with first-episode schizophrenics showed deficits only in executive attention (Orellana et al., 2012). Disorders in executive attention tasks have been related to the degree of activation of the cingulate cortex and are associated with empathy disorders in schizophrenia (Trimble and Schmitz, 2010). In fact, while sad faces activate the amygdala, additional activation of the ACC is correlated with a greater degree of sadness. The ACC may intervene in the ability to respond to the emotional expressions of others and its dysfunction may explain the significant deficit in empathy in schizophrenia (Posner and Fan, 2009).

Disorders in EFs and executive attention are considered a central element of schizophrenia and may explain the negative symptoms of the disease (Donohoe and Robertson, 2003).

## Neurocognitive Models of Schizophrenia

Schizophrenia presents a challenge to the development of cognitive models because of the extent and diversity of its symptoms that include almost all cognitive domains: perception (hallucinations), inferential thinking (delusions), fluency of thought and speech (alogia), clarity and organization of thought and language (formal thought disorder), motor activity (catatonia), emotional expression (flat affect), ability to initiate and complete goal-oriented behavior (avolition), and ability to search for and experience emotional gratification (anhedonia). Not all of these symptoms are found in a single patient and none is pathognomonic of the disease. An initial examination of the variety of symptoms may suggest that multiple brain regions are involved. Therefore, in the absence of recognizable brain lesions and known specific pathogens, researchers have explored models that may explain the diversity of symptoms with a single cognitive mechanism. Models can be divided into two groups: (i) neuroanatomical models postulating that the disorders of EFs in schizophrenia are due to the dysfunction of certain brain circuits and regions, and (ii) cognitive models postulating that certain cognitive disorders account for the symptomatology of schizophrenia. We will review some of these models.

### Connectionist model of schizophrenia

As mentioned, neuropsychological studies have shown that the more consistent and pronounced deficits of schizophrenia concern EFs, memory, and attention, which are present from the onset of the disease and perhaps in prodromal stages. Disorders in cognitive domains related to regions in direct connection with the PFC have also been described. These regions include the basal ganglia, thalamus, medial temporal lobe, and parietal lobe. It has been proposed that the deficits observed in schizophrenia may be secondary to alterations in connectivity of the cortico-subcortical or corticocortical neural networks (Pantelis et al., 2002). Thus, schizophrenia has been conceptualized as a disorder of neural connectivity involving prefronto-striato-thalamic, prefronto-temporal, prefronto-thalamo-cerebellar, and prefronto-parietal neural networks, and disconnection of these neural networks may explain schizophrenia symptoms, cognitive deficits, and neuroimaging findings (Schmitt et al., 2011). One hypothesis that attempts to unify the available evidence suggests that schizophrenia may be a disorder of the cognitive networks that involve the heteromodal association cortex, which consists of the DLPFC, superior temporal cortex, and inferior parietal cortex. These cortices are interconnected and have extensive connections with limbic and subcortical structures (Pantelis et al., 2002).

We will describe three models of the pathophysiology of schizophrenia, which postulate that dysfunction of the PFC and its connections with other regions may explain the main symptoms of the disease.

#### Schizophrenia as a disruption of the fronto-striato-thalamic system

Neuropathology, and structural and functional neuroimaging studies have shown alterations in the PFC in schizophrenia, and studies using brain magnetic resonance spectroscopy have identified abnormalities in prefrontal neurons in schizophrenia. Functional and structural changes in the basal ganglia and thalamus have also been identified (Pantelis et al., 2002). It has been suggested that disorders of EFs in schizophrenia may be associated with dysfunction of the three frontosubcortical circuits related to cognitive and behavioral control: the DLPFC, OFC, and ACC circuits. Additionally, changes in eye movements observed in schizophrenia have been related to a dysfunction of some regions of PFC (Hutton et al., 2004; Camchong et al., 2008). Dysfunctions of the cingulate and parietal circuits may mediate certain symptoms such as delusions of control (Spence et al., 1997; Pantelis et al., 2002). We will describe the three circuits involved in cognition and behavior.

##### The DLPFC-striato-thalamic circuit

Dorsolateral PFC dysfunction in chronic and FES has been the most consistent finding in functional neuroimaging studies. Ingvar and Franzen (1974) the first study was published showing that patients with chronic schizophrenia had less frontal blood flow compared with controls. This finding was termed “hypofrontality” and has been correlated with negative symptoms (Liddle et al., 1992). A series of subsequent studies of medicated and unmedicated schizophrenia patients have confirmed hypofunction or hyperfunction of the DLPFC during executive tasks (Pantelis et al., 2002; Eisenberg and Berma, 2010). Functional neuroimaging studies also show a compromised DLPFC in WM tests in schizophrenia (Pantelis et al., 2002; Kyriakopoulos et al., 2012). Most studies show altered connectivity among the DLPFC and other structures important for EFs. A recent study examines white matter integrity of the tracts connecting DLPFC/VLPFC and striatum in patients with FES, and their associations with WCST. This study propose that white matter tract abnormalities between rostral Medio Frontal Gyrus/Inferior Frontal Gyrus and striatum are present in FES and appear to be significantly associated with executive dysfunction but not with symptom severity (Quan et al., 2013). Furthermore, levels of *N*-acetylaspartate (NAA) measured by spectroscopy are reduced in the DLPFC, which implies reduced neuronal integrity associated with dysfunction in striatal dopaminergic activity (Pantelis et al., 2002; Eisenberg and Berma, 2010; Kubota et al., 2013). Finally, dysfunction of the DLPFC or its subcortical connections has been associated with deficits in executive tasks related to this region (see Disorders of Executive Functions in Schizophrenia).

##### The OFC-striato-thalamic circuit

There is some neuroanatomical evidence on this circuit being compromised in schizophrenia. A recent study revealed that schizophrenia was associated with reduced thalamocortical connectivity in the right orbitofrontal region (Kubota et al., 2013). OFC circuit dysfunction has been postulated due to clinical similarities between patients with OFC lesions and patients with schizophrenia. Both patients exhibit major changes in personality, which include irritability, disinhibition, inappropriate self-neglect, and loss of concern for others. Furthermore, both patients may excessively depend on environmental cues that manifests with automatic imitation of gestures and actions of others, or a tendency to use objects in the environment (Pantelis et al., 2002). Kanahara et al. (2013) found that the negative syndrome group of patients showed a significant decrease in regional cerebral blood flow in the right OFC compared to the non-negative group. Dysfunction of the VPFC or its subcortical connections has been associated with deficits in executive tasks related to this region (see Disorders of Executive Functions in Schizophrenia).

##### The ACC-striato-thalamic circuit

This circuit, which includes the medial PFC, is related to the hippocampus and has close connections with the DLPFC, OFC, and parietal cortex, sharing overlapping functions with these areas. The circuit participates in tasks that require initiation, motivation, selection, inhibition, and monitoring of error and conflict, handling the intentional selection of external stimuli based on their internal relevance to the organism. Information on internal relevance is provided by activity of the ACC circuit while the DLPFC is involved in the development of new strategies and selection of appropriate responses. Lesions of the ACC circuit result in akinesia, apathy, decreased ability to inhibit inappropriate responses, and deterioration in the ability to express and experience emotions. These symptoms are very similar to the negative symptoms of schizophrenia. The medial PFC is activated during tasks such as the Tower of London and Stroop, which schizophrenia patients have difficulty with (for a review, see Minzenberg et al., 2009).

Neuropathological studies have identified subtle abnormalities of the ACC in postmortem studies of schizophrenia, specifically at layers II, III, and IV (Ritter et al., 2004; Jovanovski et al., 2007; Orellana, 2009). The degree of folding of the ACC has been shown to significantly differ between first-episode and chronic schizophrenics, and normal controls (Eisenberg and Berma, 2010). Magnetic resonance imaging (MRI) researchers have reported decreased volume of the ACC, gray matter, and levels of NAA, and PET studies have shown a reduction of blood flow and cellular metabolism in the ACC in schizophrenia. The intensity of the disorganization syndrome in schizophrenia, including disorders of thinking and inappropriate affect, has been correlated with increased activity in the ACC at rest in PET (Cheng and Chan, 2005; Eisenberg and Berma, 2010; Kubota et al., 2013). Another PET study of schizophrenic patients during execution of the Stroop test showed less activation of the ACC when identifying the color in the color-incongruent condition, which requires inhibiting a dominant response (Carter et al., 1997). A disruption of frontocingulate functional connectivity has also been reported in schizophrenia during the execution of a verbal fluency task and attention task, a modified version of the Continuous Performance Test (Eisenberg and Berma, 2010). Interestingly, it has been suggested that treatments with neuroleptics are associated with a normalization of behavior and cerebral blood flow in the cingulate cortex (Digirolamo and Posner, 1996).

These different studies show that schizophrenic patients have deficits in PFC functions, which have been associated with dysfunction in the frontosubcortical circuits. As we will now see, other authors have proposed that schizophrenia may be explained by dysfunction in the connections between the prefrontal and retrorolandic cortices.

#### Schizophrenia as a disruption of the frontotemporal system

Neuropathological studies of schizophrenia have consistently shown structural abnormalities in the hippocampus (Arnold, 1997; Eisenberg and Berma, 2010). Goldman-Rakic and Selemon (1997) proposed that deficits of frontal lobe functioning, especially the WM deficit and other neuropsychological disorders, may be due to dysfunction of the circuit that connects the DLPFC with the hippocampus. In addition, regions with important hippocampal connections have been involved, such as the ACC and medial PFC. MRI and postmortem schizophrenia studies have shown volume loss in the medial temporal lobe, especially in the hippocampus, as one of the most consistent structural abnormalities (Wood et al., 1999; Kasparek et al., 2006). Endorsing this hypothesis, reduced performance of schizophrenics on the WCST has been correlated with hippocampal atrophy, which predicts the degree of prefrontal hypoactivation during the WCST (Weinberger et al., 1992; Shenton et al., 1993). Although these data suggest an alteration in the anatomical connectivity between the PFC and hippocampus, this correlation may also be explained by dysfunction of other structures such as the ventral anterior thalamic nucleus, which connects the medial temporal lobe and the PFC (Pantelis et al., 2002; Eisenberg and Berma, 2010; Kubota et al., 2013). Further studies with functional neuroimaging are needed to conclude that the reduction of cerebral blood flow in the DLPFC in schizophrenia is secondary to structural abnormalities of the medial temporal lobe (Eisenberg and Berma, 2010; Kubota et al., 2013). A variety of functional MRI studies revealed disturbed connectivity in complex hippocampal, PFC, and cerebellar-thalamic-prefrontal networks in schizophrenia during executive tasks (Schmitt et al., 2011).

Studies of memory in schizophrenia have yielded conflicting and inconclusive results on dysfunction of the medial temporal regions. Schizophrenics do not differ from controls in learning pairs of non-semantically related words, suggesting an intact medial temporal lobe, specifically the hippocampus (Nestor et al., 2005). These studies imply that verbal memory deficits in schizophrenia may be explained by impairment in strategic mnemonic processes dependent on the PFC and therefore by PFC dysfunction (Iddon et al., 1998; Pantelis et al., 2002; Christensen et al., 2006; Eisenberg and Berma, 2010). However, chronic schizophrenics exhibited impairment in tests of visual memory such as paired associate learning, a non-word visual memory test in which graphic patterns are paired with spatial locations (Rushe et al., 1999). This result suggests the existence of right hippocampal dysfunction in schizophrenia, which has been correlated with the presence of hippocampal atrophy (Pantelis et al., 2002; Eisenberg and Berma, 2010; Kubota et al., 2013).

In summary, although memory decline in schizophrenia is unquestionable, it is not entirely clear whether it is caused by medial temporal or frontotemporal lobe dysfunction, or more general cognitive dysfunction. There is a lack of empirical evidence to conclude on the real role and integrity of frontotemporal connections in schizophrenia (Pantelis et al., 2002).

#### Schizophrenia as a disruption of the frontoparietal system

In schizophrenia, abnormally low cerebral blood flow in the DLPFC is associated with decreased flow in the parietal lobe; these are highly interconnected regions (Kim et al., 2003). Consistent with right parietal lobe dysfunction, several studies have shown slight spatial neglect of the left hemispace in schizophrenia (Bustillo et al., 1997; Cavezian et al., 2011). This neglect is greater in patients with more positive symptoms and is often resolved after short periods of treatment with antipsychotics (Maruff et al., 1995). On the other hand, unlike in controls, an inverse relationship between functional activity of frontal and parietal cortices during executive tasks is not observed in schizophrenia patients. This result suggests dysfunction of the frontoparietal neurocognitive network (Perlstein et al., 2003; Rissman et al., 2004; Lesh et al., 2011). Neuroanatomical studies have also shown abnormal neuronal density in the parietal lobe and reduced parietal gray matter volume in schizophrenia (Goldstein et al., 1999; Kuperberg et al., 2003; Zhou et al., 2007). Parietal activation is reduced in schizophrenia during the execution of WM, semantic integration, and selective attention tasks (Barch and Csernansky, 2007; Weiss et al., 2007; Neuhaus et al., 2011). Finally, patients with focal lesions of the parietal cortex have symptoms similar to those observed in patients with schizophrenia such as alienation, neglect, anosognosia, and body image distortions. A compromised parietal lobe has been associated with delusions of control since only patients with this symptom have increased cerebral blood flow of parietal and cingulate cortices, suggesting dysfunction of those regions (Spence et al., 1997). In summary, parietal lobe dysfunction and inferior frontoparietal connections may exist in at least one subset of schizophrenics (Pantelis et al., 2002; Eisenberg and Berma, 2010; Kubota et al., 2013).

Dysfunctions in different networks may explain some symptoms of schizophrenia, specifically executive dysfunction. These findings are consistent with the concept that this disease is primarily a disorder of prefronto-striato-thalamic, prefronto-temporal, and prefronto-parietal connectivity.

### Cognitive models of schizophrenia

#### Cohen model

Context is considered to be the central element to understanding schizophrenia in the Cohen model. According to Cohen, the central feature of cognitive control is the ability to properly maintain and update internal representations of contextual information relevant to a specific task. Contextual representations are maintained online in an active state, and are constantly accessible and available to influence information processing. Therefore, context can be considered a component of WM and active maintenance of contextual information is critical for cognitive control. Contextual processing is associated with activity in the medial frontal gyrus and the DLPFC, and is modulated by the activity of dopamine. Dopamine serves as a gateway for PFC function, regulating access to context representations in active memory (D’Ardenne et al., 2012). This gives dopamine an important control function responsible for flexible updating of active memory in the PFC while protecting against interference (Miller and Cohen, 2001). A deficit in processing contextual information is prominent and persistent in chronic and first-episode schizophrenics, and does not occur in non-schizophrenic psychosis (Barch et al., 2001; Cohen et al., 2002; Holmes et al., 2005). Behavioral deficits in a wide range of cognitive domains may be explained by cognitive control failure due to an impaired ability to represent, maintain, and update contextual information in schizophrenics. Indeed, impairment in WM, attention, and inhibition has been related to contextual processing deficits (Perlstein et al., 2001). The Cohen model has provided a conceptual framework encompassing the psychological processes that are impaired in schizophrenia and its neurobiology. An important component of the pathophysiology of schizophrenia may be dysfunction of the dopaminergic system. Specifically, increased noise in dopamine system activity is presumed to occur in schizophrenia, leading to abnormal input to the PFC.The growing variability leads to a disturbance in updating and maintaining contextual information in WM. This theory postulates a single neurobiological mechanism for these deficits involving dysfunctional interactions between the dopaminergic neurotransmitter system and the PFC (Braver et al., 1999; Barch, 2005; MacDonald et al., 2005).

#### Frith model

Chris Frith, applying cognitive psychology to schizophrenia, has divided the symptoms of this disease into three dimensions: (a) “intentional conduct disorder” that leads to symptoms such as inappropriate behavior, perseveration, and abulia; (b) “self-monitoring disorders” that lead to auditory hallucinations, thought insertion, and delusions of control; and (c) “disorders in evaluating others’ intentions” that lead to symptoms such as delusions of persecution, delusions of reference, illogical speech, and third-person auditory hallucinations. The first dimension may produce the so-called negative symptoms of schizophrenia and the other two may lead to the positive symptoms of schizophrenia. Frith argues that the three dimensions are associated with a more general underlying mechanism: a disorder of consciousness or self-awareness that impairs the ability to think with meta-representations (i.e., representations of mental states) (Frith, 1995a,b, 1996).

Disorders of intentional behavior may result from dysfunction in the ability to represent actions directed toward a goal. Negative and disorganized symptoms may be explained by impaired access to goals in terms of Norman and Shallice’s SAS. This leads to a fundamental defect in the generation of intentional activity, manifested in impaired ability to: (a) form desired intentions based on current goals, (b) link goals and actions to initiate or terminate an activity, and (c) inhibit behaviors unrelated to a goal (Weinberger and Berman, 1998).

Experimental evidence in favor of this model is based on PET studies of participants with or without schizophrenia. To study the representation of intentional actions, participants were asked to perform self-generated actions and the correct answer was not obvious from the context, such as in a verbal fluency test or choosing the flick of a finger. In participants without schizophrenia, prefrontal regions were activated, whereas schizophrenics demonstrated less activation of frontal regions and greater activation of temporal regions. By slowing performance speed on the verbal fluency task, normalization in frontal activation was observed with persistent abnormality only in the temporal lobe in schizophrenics (Frith et al., 1995; Spence et al., 2000). Studying the correlation between blood flow in frontal and temporal regions suggested altered functional connectivity (Stephan et al., 2009). Frith proposes that abnormal functional integration of brain processes occurs in schizophrenia (Andreasen, 1997; Stephan et al., 2009).

On the other hand, according to this model, hallucinations in schizophrenics may be explained by impaired recognition of internal speech as their own and attribution of this speech to another person, reflecting a deficit in self-monitoring. McGuirre and collaborators studied brain activation during a task that simulates auditory hallucinations–participants were required to complete a sentence and imagine that the response was said by the voice of another person. Activation of speech production and perception regions, such as Broca’s area, the SMA, and the left medial and superior temporal regions, was observed in participants without schizophrenia. Schizophrenic patients with hallucinations, unlike those without hallucinations, showed decreased activation in areas associated with supervision of speech, namely the left medial temporal circumvolution and the SMA (McGuire et al., 1996). In other studies, Frith and collaborators examined cerebral blood flow in schizophrenics while experiencing auditory hallucinations and found activity mainly in subcortical regions (thalamus and striatum), limbic and paralimbic regions (ACC and parahippocampal gyrus), and the cerebellum. Frith postulates that activity in subcortical regions may generate or moderate hallucinations, whereas content (auditory or tactile) is determined by activity in specific neocortical regions (Frith, 1997; Johns et al., 2001; Shergill et al., 2005; Fletcher and Frith, 2009).

## Summary and Future Directions

The various impairments in cognitive functions in schizophrenia, including executive dysfunction or behavioral control, may be due to dysfunction of connectivity and communication of micro- and macro-circuits in the cortico-subcortical prefronto-striato-thalamic network, or corticocortical, prefronto-temporal, and prefronto-parietal neural networks (Evans et al., 1997; Lesh et al., 2011). Key brain regions that show postmortem and *in vivo* evidence of disarray in schizophrenia are important for executive functioning, and are physiologically abnormal during executive challenge in patients, evidenced by characteristically aberrant interactions and high susceptibility to variation in putative schizophrenia risk genes. DLPFC dysfunction and aberrant functional connectivity can lead to: (i) increased VLPFC involvement in executive circuitry, (ii) increased dysfunction of the ACC and inferior parietal lobe, and reduced coupling with the DLPFC, (iii) increased impairment in suppression of medial temporal activity during certain executive challenges, (iv) prefrontal disinhibition of mesostriatal dopaminergic signaling, and (v) reduced thalamo-frontal cooperativity. These consequences not only form a complex landscape of circuit changes in schizophrenia, but selected subsets also create quantifiable links to emerging molecular footprints of genetic predisposition to psychosis. Systematic work is needed to better characterize the dynamics of these systems-level abnormalities in response to particular executive task demands, treatment interventions, and genetic environments in children at risk for schizophrenia, adolescents at ultra-high risk for schizophrenia, first-episode schizophrenics, and chronic schizophrenics (Eisenberg and Berman, 2010; Lesh et al., 2011).

**Figure 3:**
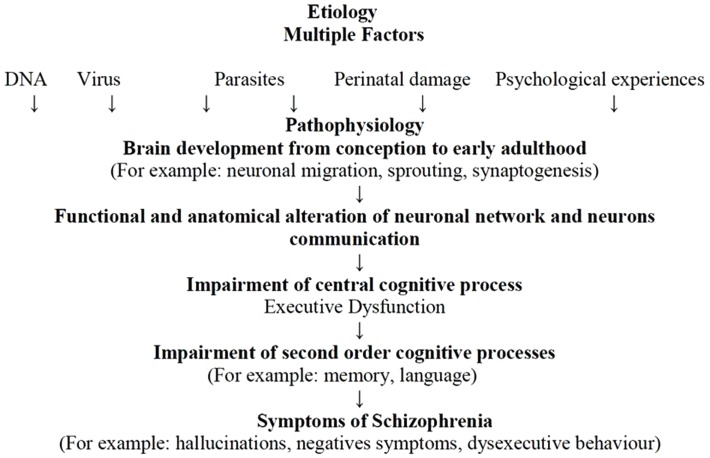
**The Andreasen model adapted from Andreasen (2000)**.

The etiologies of schizophrenia are multifactorial – an accumulation of genetic and non-genetic influences from which stems the possibility that exposure to different pathogens may produce brain lesions and may combine with environmental factors, especially psychological factors, to affect neuronal plasticity. This process would produce alterations from pregnancy through to adolescence in neurodevelopment (i.e., processes of neuron formation), synaptogenesis, neuronal pruning, apoptosis, and neuronal modifications induced by activity, which would lead to anatomical and functional alterations in neuronal connectivity and communication. As previously discussed, structural and functional abnormalities of the brain in schizophrenia involve multiple areas (i.e., frontal and temporal cortices, thalamus, hippocampus, basal ganglia, and cerebellum), suggesting that schizophrenia may be a disease of neuronal connectivity (Andreasen, 1997) (cf. Figure 3).

Disorders of communication and connectivity in neuronal circuits may produce disturbances in EFs linked to the PFC and related structures. Impairments in DLPFC function, its interaction with other brain regions (e.g., parietal cortex, thalamus, and striatum), and the influence of neurotransmitter systems (e.g., such as dopamine, GABA, and glutamate) explain the inability to actively represent goal information in WM, which is needed to guide behavior. This inability represents a common mechanism contributing to cognitive impairments across a range of domains in that EF disorders may cause impairment in second-order cognitive processes such as memory, language, or emotion (Barch and Ceaser, 2012). Alteration in first- and second-order cognitive processes may eventually produce schizophrenia symptoms such as hallucinations, negative symptoms, and dysexecutive behaviors.

Schizophrenics may demonstrate the most significant alterations in EFs, a first-order cognitive process. This may consequently lead to cognitive disorders in second-order functions, memory, or social language. Executive dysfunction is manifested in dysexecutive behaviors, which may account for most problems patients face in their daily lives. Better comprehension of the association between negative symptoms and dysexecutive behaviors can help us understand the social adjustment disorders of schizophrenia. Further studies are needed to explore the evolution of dysexecutive disorders over the course of the disease, and its response to pharmacological and non-pharmacological treatments. It is also important to investigate the longitudinal course of executive dysfunction in schizophrenia because this dysfunction is already present in children who later develop schizophrenia, and maturation of EF extends into young adulthood.

Neuroimaging has played a central role in providing abundant evidence of structural and functional connectivity abnormalities in schizophrenia. In recent years, our understanding of how schizophrenia affects brain networks has been greatly advanced by attempts to map the complete set of inter-regional interactions comprising the brain’s intricate web of connectivity (i.e., the human connectome). Imaging connectomics refers to the use of neuroimaging techniques to generate these maps, which has enabled relatively comprehensive mapping of brain network connectivity and topology in unprecedented detail when combined with the application of graph-theoretic methods. Researchers have since applied these techniques to the study of schizophrenia, focusing principally on MRI research. The published findings suggest that schizophrenia is associated with a widespread and possibly context-independent functional connectivity deficit upon which context-dependent alterations associated with transient states of hyper- and/or hypoconnectivity are superimposed. In some cases, these changes in inter-regional functional coupling dynamics can be linked to measures of intra-regional dysfunction. Topological disturbances of functional brain networks in schizophrenia point to reduced local network connectivity and modular structure as well as increased global integration and network robustness. Some, but not all, of these functional abnormalities appear to have an anatomical basis, though the relationship between the two is complex. By comprehensively mapping connectomic disturbances in patients with schizophrenia across the entire brain, this work has brought light to the highly distributed character of neural abnormalities in this disorder, and the potential functional consequences that these disturbances entail (Fornito et al., 2012).

Several avenues of research promise to provide invaluable insights into the pathophysiology and ultimately, targeted treatment of this devastating illness. To address accumulating evidence of the genetic heterogeneity underlying the disorder and concomitant variability in psychopathological and neuropsychological profiles, all of which may have contributed to apparent inconsistencies in the literature, more extensive genetically, clinically, and cognitively stratified neuroimaging studies are necessary. Likewise, longitudinal studies directed at understanding how naturalistic cognitive remediation and pharmacologically induced fluctuations in executive network function are essential to assess the stability of circuit perturbations in schizophrenia over the course of the illness and treatments (Eisenberg and Berma, 2010).

## Conflict of Interest Statement

The authors declare that the research was conducted in the absence of any commercial or financial relationships that could be construed as a potential conflict of interest.
